# Anesthesia and Intensive Care Management in a Pregnant Woman with PRES: A Case Report

**DOI:** 10.1155/2012/745939

**Published:** 2012-07-05

**Authors:** Ismail Demirel, Ayse Belin Ozer, Mustafa K. Bayar, Salih Burcin Kavak

**Affiliations:** ^1^Department of Anaesthesiology and Reanimation, Faculty of Medicine, Firat University, 23119 Elazig, Turkey; ^2^Department of Obstetrics and Gynecology, Faculty of Medicine, Firat University, 2311 Elazig, Turkey

## Abstract

Posterior reversible encephalopathy syndrome (PRES) is a temporary condition that is diagnosed clinically, neurologically, and radiologically. Its symptoms vary, and nonspecific headaches, confusion, impairment of consciousness, nausea, vomiting, and visual impairment may occur. Acute hypertension often accompanies these symptoms. Patients can also suffer from convulsions, cortical visual impairment, and coma. Diagnosis can be difficult due to focal neurologic signs. Nevertheless, knowing the clinical risk factors can lead to the right diagnosis. It has been reported that this condition may also occur during organ transplantation, immunosuppressive treatment, and autoimmune diseases and chemotherapy, and also patients with eclampsia. In this paper, a 21-year-old, 31-week pregnant woman, who had been diagnosed with PRES and thanks to early diagnosis and treatment had fully recovered and discharged from the intensive care unit, is presented, and the relevant literature is discussed.

## 1. Introduction

Originally described in 1996 as a disease of the posterior cerebrum PRES is the clinical syndrome of vasogenic edema in the central nervous system. It is a cerebral vascular autoregulation deficiency resulting from sudden changes in blood pressure. It is characterized by headaches, generalized seizures, vision impairment, lethargy, confusion, stupor, changes in mental status, and focal neurologic signs. Diagnosis is made by piecing together clinical and radiological findings [1]. The etiologic factors of this condition are hypertensive encephalopathy, eclampsia/preeclampsia, drug use, renal disease (acute and chronic), thrombotic thrombocytopenic purpura, systemic lupus erythematosus, hemolytic uremic syndrome, and diseases that cause immune system deficiency (leukemia, lymphoma) [2, 3]. With early diagnosis and treatment, patients can recover clinically in a few weeks. If not treated in time, the condition can get worse, resulting in cerebral ischemia, infarcts, and even death. As there are no clinically specific signs for the syndrome, it can often be confused with other clinical conditions, leading to unnecessary and/or wrong treatments [4].

Preeclampsia is a syndrome characterized by hypertension, edema, proteinuria, and the activation of coagulation mechanisms after the 20th week of pregnancy. Many hypotheses have been suggested regarding the mechanism of the disease process, and it is stated that the condition occurs due to issues regarding placental location and trophoblastic invasion. Severe systemic vasospasm, which is the most significant physiological change in the patients, causes reduced perfusion of all organs. Other reasons for the hypoperfusion are hemoconcentration and third-space loss. Moreover, inappropriate endothelial activation and increased inflammatory response can be observed [5]. If preeclampsia is not treated or does not respond to treatment, it can lead to a more serious condition, that is, eclampsia condition that covers all the symptoms of pre-eclampsia with the addition of seizures. PRES can occur during pregnancy and in the postpartum state. Radiological findings of PRES resulting from pregnancy are identical to the radiological findings in other etiologic causes of PRES. The most important differential diagnosis that one must keep in mind about these patients is dural sinus thrombosis.

Magnetic resonance imaging (MRI) typically shows hyperintensity in the bilateral parieto-occipital areas in sequences T2A and FLAIR, consistent with vasogenic edema. In atypic involvements of PRES, frontal lobe, basal ganglia, brain stem, and deep white matter are affected [6, 7]. Recurrent attacks of PRES are mainly related with eclampsia, and their incidence is proportional to recurrent eclampsia [8]. With this paper, we wanted to draw attention to the fact that an eclamptic patient 31 weeks pregnant, who developed PRES, was treated in the intensive care unit after an early diagnosis and was discharged fully recovered, and that there are more than one disease entity that may result in PRES.

## 2. Case Report

**Figure 1:**
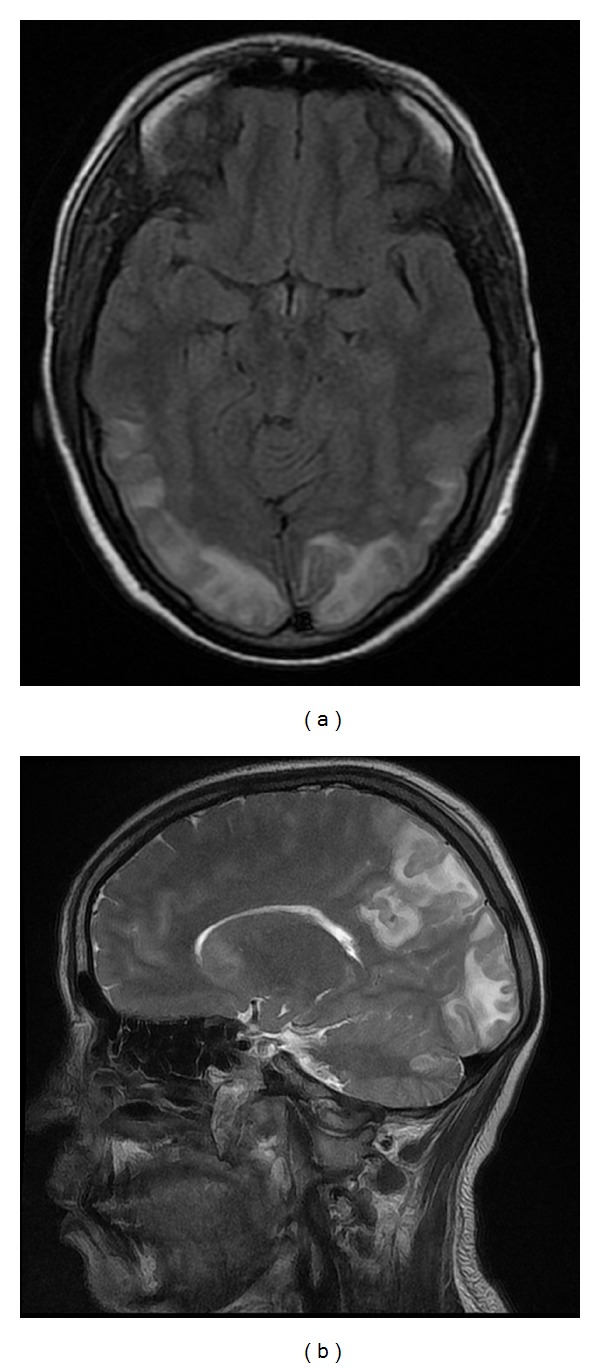
The patient MRI findings in postoperative first day.

**Figure 2:**
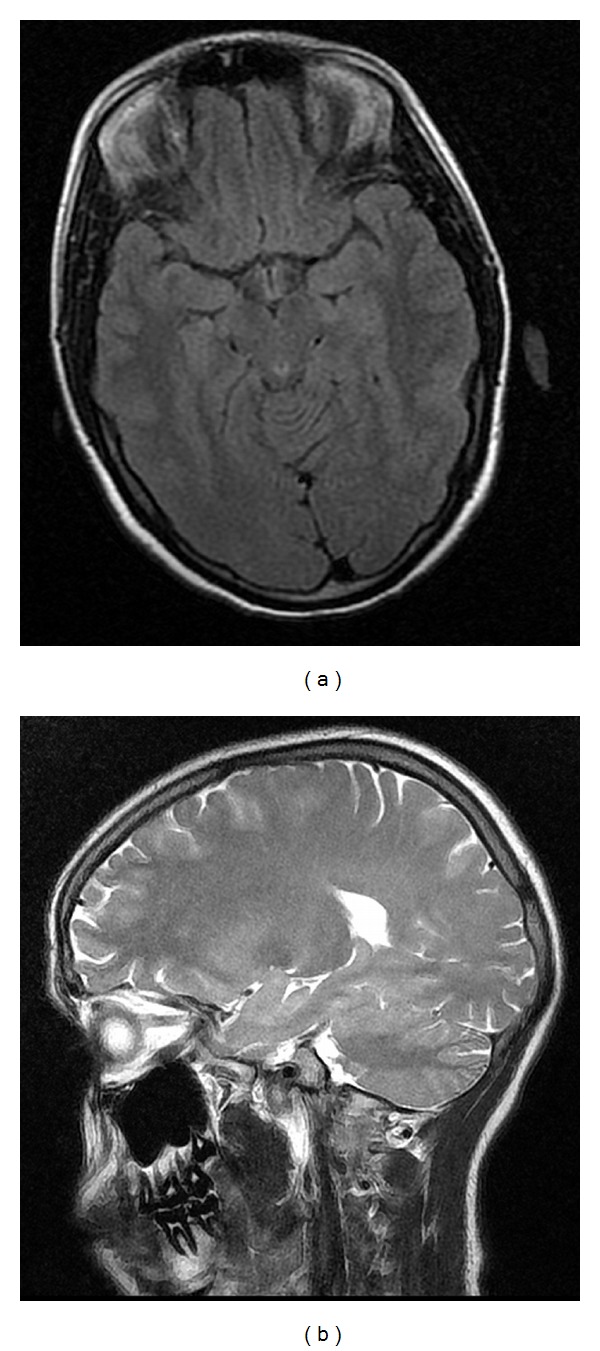
The patient MRI findings in one month later.

A 21-year-old, 31-week pregnant, gravida 1, para 0 case was confused with a moderately poor general condition and was admitted to the obstetric clinic. When the patient came to the hospital, her arterial blood pressure was 210/130 mmHg, and anamnesis from her relatives revealed that she had a generalized seizure 1 hour prior to admission. On bed-side ultrasound, normal amniotic fluid volume and one viable fetus with a body size consistent with 29 weeks were found. The patient had 4+ proteinuria and was diagnosed as eclamptic and given a loading dose of magnesium sulphate (4 grams). Three minutes after magnesium sulphate was administered, the patient had another generalized convulsion and was given diazepam (10 mg i.v.). The patient had respiratory distress and was intubated orotracheally and taken to the operating room for an emergency Cesarean section. Thiopental sodium (250 mg, Pental) and cisatracurium (4 mg, Nimbex) was administered for induction of anesthesia followed by 50% O_2_ + 50% N_2_O and 0.75% MAC isoflurane. One viable female baby in breech presentation was delivered by Cesarean section. The baby's birth weight was 1100 gr and height was 36 cm with a 1-minute APGAR score of 7. After the operation, the patient, still entubated, was taken to the intensive care unit, and mechanical ventilation was initiated (SIMV, f: 12/min, FiO_2_: 60, TV: 500 mL, I : E = 1 : 2). In the intensive care unit, her blood pressure tended to increase (180/110 mmHg) so nitroglycerin (10 *μ*g/kg/h) and magnesium sulfate (2 gr/h) infusion was continued. The patient was administered dexamethasone (32 mg/day) and an oral antihypertensive amlodipine (Norvasc 10 mg/day). As she regained consciousness and spontaneous breathing, the patient was extubated. But 15 min after extubation, the patient had another convulsion and was reintubated. Biochemical values of the case were as follow: Hb: 13.5 gr/dL, Htc:  %37, WBC: 16590/mm^3^, PLT: 50.000/mm^3^, AST: 375 U/L, and ALT: 183 U/L, LDH: 1213 U/L, and her coagulation parameters were Prothrombin time (PT): 52,1 secs, activated prothrombin time (APTT): 25,2 secs, INR: 1,44. With these findings, the patient was diagnosed as having HELLP syndrome and eclampsia, and neurologic examination showed no lateralization. The patient was administered Thiopental sodium (100 mg bolus followed by a continuous infusion of 250 mg/h i.v.), and her convulsions were reduced considerably. Therefore, first day after surgery thiopental sodium was stopped, and after approximately 6 hours, the patient was extubated. Magnesium sulfate infusion was continued. The patients, postoperative first-day MRI showed increased intensity lesions on sequences T2, FLAIR, DAG, and ADC in bilateral basal ganglia, at the level of centrum semiovale on the frontal areas, bilateral parietotemporal, occipital regions, and left cerebellum (Figure 1). The patient was diagnosed as having PRES. The patient was treated with magnesium sulfate for 48 hours, and her platelet counts were 44,000/mm^3^ on the first day after the operation. Her liver enzyme and LDH levels were elevated. As her general condition improved, the patient was taken to the obstetric clinic on the second postoperative day. Her liver enzyme and LDH levels started to decrease starting from the second postoperative day. Her platelet counts were 97,000/mm^3^ on the second day after surgery. Her drains were removed on the third day after the surgery and sutures were removed on the 7th postpartum day. The patient was discharged with suggestions after that. Her MRI was taken after a month and was totally normal (Figure 2).

## 3. Discussion

PRES is a clinical condition that causes neurological symptoms such as variable consciousness, seizures, and vision impairment, and its symptoms and imaging findings are generally reversible. Many diseases such as hypertension, eclampsia/preeclampsia, immunosuppresive drugs (cyclosporine), various antineoplastic agents, hypercalcemia, thrombocytopenic syndromes, Henoch-Schönlein purpura, hemolytic uremic syndrome, systemic lupus erythematosus, amyloid angiopathy, and renal failure can cause PRES. The pathophysiology of PRES is unclear. The generally accepted theory is edema formation in subcortical white matter as a result of the extravasation due to sudden changes in blood pressure and/or toxins that damage the endothelium and disrupt the blood brain barrier. Another opinion is that vasospasm is the underlying cause with resulting cytotoxic edema in affected areas [9].

The typical MRI findings of PRES are symmetric increases of the signals in the bilateral parieto-occipital areas, subcortical white matter of bilateral frontal and temporal lobe, posterior segments, and sometimes the cortex in sequences FLAIR and T2. In diffusion MRI images, increased diffusion consistent with vasogenic edema is detected. Lesions due to vasogenic edema are reversible. However, in some cases, lesions with reduced diffusion due to cytotoxic edema can be determined, and these lesions generally heal leaving a sequel. In atypic presentations, high-contrast lesions and hemorrhage can be seen in the thalamus, basal ganglia, brain stem, and cerebellum [9]. Our patient had increased intensity of the lesions on sequence T2 in the bilateral basal ganglia, at the level of centrum semiovale in the frontal areas, bilateral parietotemporal, occipital regions and left cerebellum.

PRES is a condition that rapidly responds to aggressive treatment. Clinical and radiological findings are reversible. Its clinical symptoms are similar to many neurological diseases. The following disorders must be considered in differential diagnosis: demyelinating diseases, basilar artery embolism, and venous sinus thrombosis. The first step in treatment must be correcting the underlying etiological factors. Preventing hypertension and other triggers (cytotoxic conditions, immunosuppressive drugs, sepsis, and the like) is the key. For our patient, the etiology was thought to be eclampsia as a result of her pregnancy and encephalopathy because of disturbed blood pressure autoregulation. Her condition rapidly changed for the better once the convulsions stopped, and her blood pressure was normalised. This is why the condition is called posterior “reversible” encephalopathy syndrome [10]. Oral and i.v. antihypertensive agents, sedative hypnotics, and diuretics can be used to treat the hypertension [11–14]. Our patient had a generalized seizure on her 31st week of pregnancy, was admitted to the hospital, was started on magnesium sulfate infusion, and after her second generalized seizure was given additionally diazepam and was taken for a Cesarean section after being orotracheally intubated. When the patient had another generalized seizure in the intensive care unit after the operation, the patient was reintubated orotracheally and was administered thiopental sodium in addition to magnesium and i.v. antihypertensive agents. The patient was diagnosed with PRES that responded to early treatment. Magnesium sulphate is widely used for preeclamptic patients to prevent eclamptic seizures. Generally, 4–6 gr i.v. is administered in the first 20 minutes continued with 1-2 gr/h. During the treatment, the patient's deep tendon reflexes, respiratory rate, heart rate, blood pressure, and urine output must be monitored closely. Magnesium shows its central anticonvulsant effect by antagonizing glutamate's attachment to n-methyl-d-aspartate (NMDA) receptors. It reduces postsynaptic membrane sensitivity and muscle membrane stimulation by decreasing the calcium transport in the presynaptic space and the release of acetylcholine. During general anesthesia, even in patients taking standard doses of magnesium sulphate, the effect of neuromuscular blocking agents can be potentialized, and their duration can be prolonged [15, 16]. Drugs with low biotransformation rates (like isoflurane), low renal clearance, short half-life, and low active metabolites (like atracurium) must be chosen for general anesthesia. Nitroglycerin and magnesium sulphate are suggested to prevent hypertensive attacks that can occur during the induction of anesthesia [17, 18]. We used isoflurane for anesthesia maintenance and cisatracurium as a neuromuscular blocking agent.

Striano et al. retrospectively analyzed 3000 cases, who were reviewed in order to identify subjects with a clinical history of PRES, in their study, and came across 8 cases with PRES. Five of these cases had eclampsia, and 2 of them had postpartum eclampsia [19]. Although magnesium sulphate is routinely used in eclampsia, it is reported that prophylactic use of magnesium sulphate has limited effect in preventing postpartum eclampsia [20]. After PRES did occur, magnesium sulphate can be inadequate for the treatment of postpartum eclampsia. Magnesium sulphate suppresses the seizures by regulating the Ca^++^-Mg^++^ metabolism and relaxing the muscles. It decreases cerebral edema formation after brain injury an has neuroprotective and anticonvulsant property. In addition to this, there are cerebral ischemic and edematous areas in PRES that cause the seizures. Antiedematous, antiepileptic, and sedative hypnotic must be added to treatment for cases like this. In patients with PRES as a result of Burkitt's lymphoma, there can be seizures that do not respond to dexamethasone, phenytoin, and valproic acid. Thiopental sodium is successfully used to treat this kind of generalized seizure [21]. In our patient, generalized tonic-clonic seizures occurred while she was under the treatment of magnesium sulphate, and with the addition of thiopental sodium infusion, her condition rapidly regressed. Furosemide and dexamethasone were additionally given.

In conclusion, PRES is a condition that is caused by multifactorial etiological factors, and its clinical presentation may vary. The diagnosis can be confirmed by radiological imaging studies. With early diagnosis, the syndrome may reverse without sequels. If mental status disorders and generalized seizures in the prepartum period, during the delivery, and in the postpartum period are present in the presence or absence of preeclampsia/eclampsia, must suspect the possibility of PRES and investigated. Our patient with PRES secondarily to eclampsia on the 31st week of pregnancy was diagnosed early and treated in the intensive care unit with good result. This paper is intended to remind critical care specialists of the etiology and differential diagnosis of PRES, which is a relatively rare condition.
